# Socio-environmental factors and diarrheal diseases in under five-year old children in the state of Tocantins, Brazil

**DOI:** 10.1371/journal.pone.0196702

**Published:** 2018-05-16

**Authors:** Volmar Morais Fontoura, Iolanda Graepp-Fontoura, Floriacy Stabnow Santos, Marcelino Santos Neto, Hanari Santos de Almeida Tavares, Maria Onice Lopes Bezerra, Marcela de Oliveira Feitosa, Adriano Figuerêdo Neves, Jesuane Cavalcante Melo de Morais, Luiz Fernando Costa Nascimento

**Affiliations:** 1 Department of Nursing, State University of Tocantins, Augustinópolis, Tocantins, Brazil; 2 Pos-Graduate Program in Environmental Sciences, University of Taubaté, Taubaté, São Paulo, Brazil; 3 Department of Nursing, Federal University of Maranhão, Imperatriz, Maranhão, Brazil; Uniformed Services University of the Health Sciences, UNITED STATES

## Abstract

**Background:**

Diarrhea is a waterborne disease that affects children, especially those under 5 years of age. The objective of this study was to identify the spatial patterns of distribution of diarrheal disease in under 5-year-old children in the State of Tocantins, Brazil, from 2008 to 2013.

**Methods:**

Geoprocessing tools were used to carry out an epidemiological study, to prepare thematic maps in the TerraView 4.2.2 *software* based on secondary data. General indicators of the disease, presence of spatial dependence through the Global Moran’s Index (I) and the Spatial Association Index (LISA) were described.

**Results:**

There were 3,015 cases of under 5-year-old children hospitalized for diarrhea, with an average annual rate (AAR) of 4.10/1,000 inhabitants (inhab.). Among the main characteristics were: increasing rates in under 1-year-old children (6.16 to 9.66/1,000 inhabitants); children aged 1 to 4 full years (63%); males (55%); 8 deaths of under one-year-old children (75%); county of Araguaína (67%); incidence in the county of Nazaré (63.97/1,000 inhab.); prevalence and incidence in the Araguaína microregion (45%, AAR 9.38/1,000 inhab.). The presence of a cluster with spatial autocorrelation was found in the Araguaína microregion, which was statistically significant (I = 0.11, *p*-value < 0.03), with priority of intervention (Moran Map).

**Conclusions:**

There was an increase in the number of hospitalizations for diarrhea in under 5–year-old children in the state of Tocantins. The spatial analysis identified clusters of priority areas for measures of maintenance and control of diarrheal diseases.

## Introduction

Environmental and climatic problems, poor quality of life of populations coupled with lack of basic sanitation, and difficult access to health facilities have directly contributed to the increase of cases of waterborne diseases, among them diarrhea [1–4]. This is most evident in underdeveloped and developing countries. *Vibrio Cholerae*, *Shigella* [5–7], *entamoeba histolytica*, *giardia lamblia* [8], *rotavirus* and *Escherichia coli* are present in most diarrhea outbreaks in developing countries [2,3,9–12,8], in which rotavirus is the main etiologic agent [13]. Approximately 2 million cases of people with diarrheal diseases are reported every year, of which 1.9 million are under 5–year-old children that evolve to death in developing countries [3,4,9,11,14]. One out of every five deaths among children is caused by diarrhea [5], and 50% of the cases are caused by rotavirus in countries such as India, Pakistan, Ethiopia, Nigeria and Congo Republic [15]. The incidence of diarrhea in people older than 5 years is lower, although elderly people are also vulnerable to the disease [16].

In Brazil, the group most affected by diarrheal diseases is under 5–year-old children, among which the most vulnerable are those under 1 year of age. Between 2000 and 2009, 80% of the 24 thousand deaths registered in DATASUS were under 1-year-old children [14,17]. In the Northern region, diarrhea is considered the 8th cause of infant mortality [18]; 40% of hospitalizations are caused by rotavirus and its transmission has a seasonal character [13], varying according to climatic events, seasons and geographical location [14,18], basic sanitation, adequate garbage disposal, and hygiene [19]. Infant mortality has declined considerably in Brazil in the last 10 years, especially due to the decrease in the number of deaths caused by diarrhea [8].

Diarrhea has multiple pathways of transmission [5] but the most common is low availability of water and use of untreated pathogenic water [9,18], garbage thrown in the home surroundings or vacant lots, lack of basic sanitation, and poorly built houses with lack of basic infrastructure [10,16,20]. The pathogens invade the intestinal walls causing hydroelectrolytic changes [9]. Clinical manifestations usually have a sudden onset, with watery bowel movements or feces with decreased consistency [9,14] and usually greenish color and foul odor. These manifestations last 24 hours or longer and may be accompanied by fever, vomiting, abdominal pain, loss of appetite and severe dehydration and/or malnutrition with an impact on the physical and intellectual development of the affected child or individual [9,12,14,15,17], mainly due to suppression of growth hormone production [19]. The main causes of infection are social inequality, poor hygiene, low socioeconomic status, overpopulation, low birth weight, low level of knowledge of mothers, lack of adherence to breastfeeding, poor conditions and difficult access to public health services [2,3,9–12,21], with an increase in contamination rates after hydrometeorological catastrophes and events such as floods [18].

The use of spatial analysis and Geographic Information System (GIS) tools allows the creation of maps that help in the analysis and better understanding of spatial patterns of epidemiological data distribution, making it possible to detect areas of greater risk, as well as associated factors to indicate points with greater and smaller need for intensification and/or prioritization of control measures [20,22–25]. Although geographic distribution models have been widely applied to analyze the spatial distribution of other diseases such as visceral leishmaniasis [20,23,25,26], dengue [22], leprosy [27], neonatal mortality [24] and malaria [28], they have been little used in the context of diarrheal diseases [1].

The objective of this study was to examine the spatial patterns of distribution of diarrheal disease in under 5-year-old children in the state of Tocantins, Brazil, between 2008 and 2013, in order to identify areas with high rates of diarrheal diseases and also to observe the possible global and local autocorrelation of the occurrence of diarrhea with factors associated with sanitation and garbage disposal.

## Methods

### Study area

**Fig 1:**
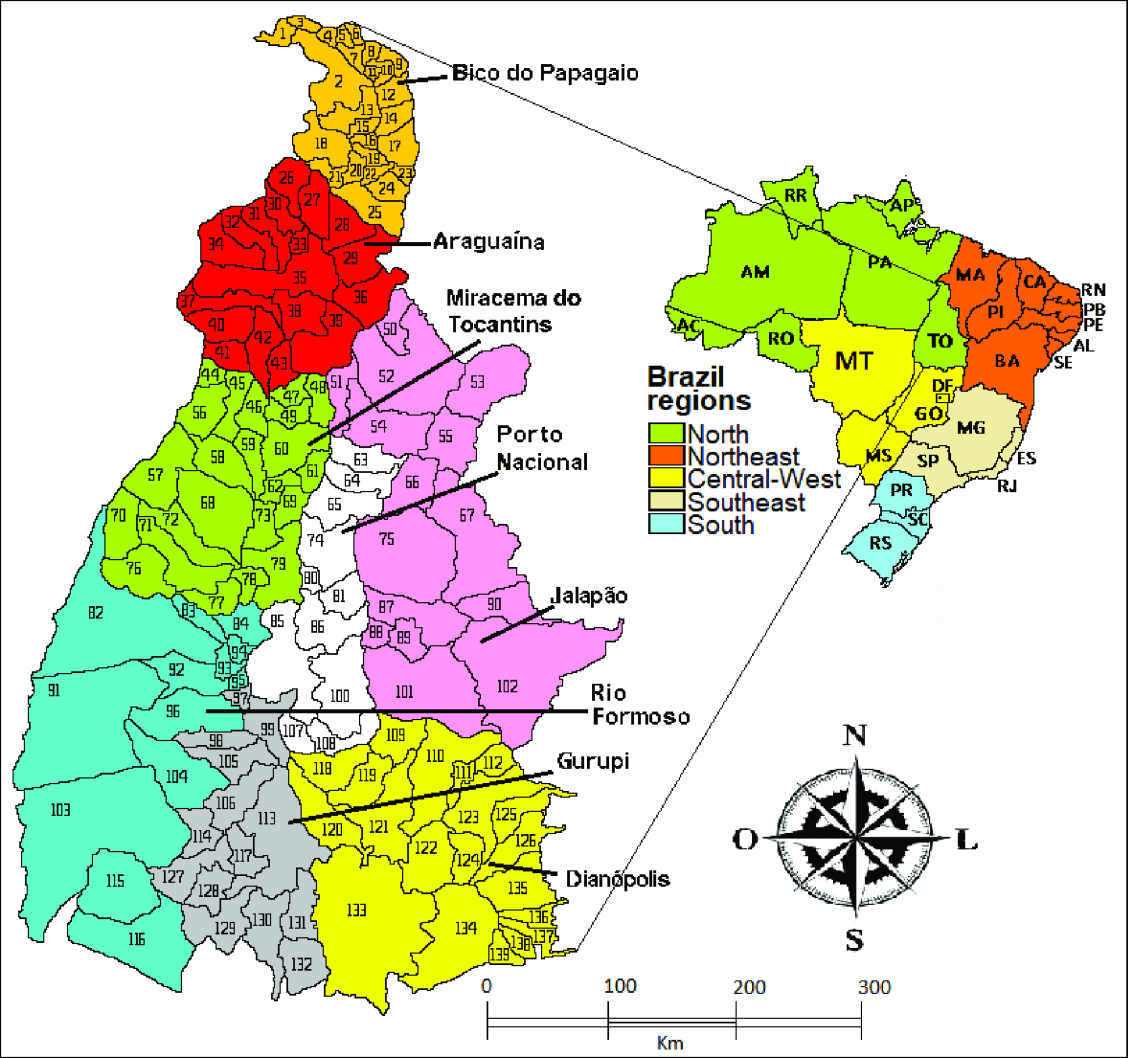
Location of the state of Tocantins in Brazil, and division into microregions and counties numbered in growing sequence from North to South.

The state of Tocantins is located in the northern region of Brazil and has 139 counties. Its population in the last census in 2010 was 1,383,445 inhabitants, 122,709 of which were under 5–year-old children (<1 year old: 23,718, 1 to 4 full years of age: 98,991). Its territory covers 277,620 km^2^. Fig 1 shows the map of Tocantins divided into microregions and the counties numbered in a growing sequence from North to South, and its location in Brazil [23,25].

An exploratory study with an ecological design based on secondary data of morbidity and mortality due to diarrheal diseases in under 5-year-old children was conducted. The choice for age groups of under 1 year of age and 1 to 4 full years of age is based on the age ranges available in the Department of Informatics of the Unified Health System (DATASUS) of the Brazilian Ministry of Health (BMOH), which are the age groups most vulnerable to complications due to diarrheal diseases. The group under 1 year of age was highlighted because this age is the most vulnerable period to diseases among children, especially in the case of diarrheal diseases.

The spatial distribution of cases of hospitalization for diarrhea from 2008 to 2013 was studied and analyzed in terms of rates in the 139 counties of the state of Tocantins, Brazil, by place of residence, and according to the year of service. To avoid bias, the location of cases of diarrhea was obtained according to hospital morbidity by place of residence of children, considering that, regardless the place of residence, children with diarrhea in a state of important dehydration are usually taken to the nearest hospitals, either directly by the parents or transferred from Family Health Strategy (FHS) units according to the Integrated Care for Childhood Illness (ICCI) protocol [29].

Data were obtained from the DATASUS of the BMOH, which are public domain data, available free of charge. All cases of diarrhea reported per year were included in the study, according to the list of morbidities of the *International Classification of Diseases 10th Revision (ICD-10*) and Related Health Problems, "A09—Diarrhea and gastroenteritis of presumed infectious origin" (vertical: total cases, gender, color/race, age group, deaths, 10 counties with the highest rates, microregions according to IBGE and according to the years in horizontal [Table 1]: http://tabnet.datasus.gov.br/cgi/deftohtm.exe?sih/cnv/nrto.def), obtained through the completion of standardized and notified forms filled out by health professionals. The analysis was based on two determinant indicators of infant diarrhea in Tocantins based on DATASUS data, namely, garbage "dumped on vacant areas or in the home environment" (http://tabnet.datasus.gov.br/cgi/deftohtm.exe?ibge/cnv/lixto.def), and sanitation characterized by "Lack of sanitary installations" (http://tabnet.datasus.gov.br/cgi/deftohtm.exe?ibge/cnv/santo.def), according to the 2010 Demographic Census. Population data were obtained from the Brazilian Institute of Geography and Statistics (IBGE: https://sidra.ibge.gov.br/tabela/3175), according to the 2010 National Population Census.

**Table 1:** Epidemiological characteristics of hospitalizations for diarrhea in under 5-year-old children in the state of Tocantins, 2008–2013.

Variables	2008			2009			2010			2011			2012			2013			Total		
**Gender**	n	%	Rate	n	%	Rate	n	%	Rate	n	%	Rate	n	%	Rate	n	%	Rate	n	%	AAR
**Total**	349	12	2.84	345	11	2.81	566	19	4.61	499	16	4.07	597	20	4.87	659	22	5.37	3015	-	4.10
**Male**	187	54	3.01	179	52	2.88	310	55	4.99	282	57	4.54	324	54	5.22	365	55	5.88	1647	55	4.42
**Female**	162	46	2.67	166	48	2.74	256	45	4.23	217	43	3.58	273	46	4.51	294	45	4.85	1368	45	3.76
**Color of the skin/ethnicity**																					
**Ign/white** *	268	-	-	263	-	-	179	-	-	163	-	-	102	-	-	130	-	-	1105	-	-
**White**	12	15	0.34	6	7	0.17	52	13	1.49	55	16	1.58	85	17	2.44	96	18	2.76	306	16	1.46
**Black**	0	0	0.00	1	1	0.16	5	1	0.78	4	1	0.62	6	1	0.93	7	1	1.09	23	1	0.71
**Brown**	62	76	33.05	72	88	38.38	301	78	160.45	241	72	128.46	386	78	205.76	379	72	202.03	1441	75	128.02
**Yellow**	4	5	0.05	1	1	0.01	2	1	0.03	3	1	0.04	3	1	0.04	1	0	0.01	14	1	0.03
**Indigenous**	3	4	1.38	2	3	0.92	27	7	12.40	33	10	15.15	15	3	6.89	46	9	21.12	126	7	9.64
**Age group**																					
**Under 1 year**	146	42	6.16	147	43	6.20	187	33	7.88	199	40	8.39	229	38	9.66	195	30	8.22	1103	37	7.75
**1 to 4 full years**	203	58	2.05	198	57	2.00	379	67	3.83	300	60	3.03	368	62	3.72	464	70	4.69	1912	63	3.22
**Deaths**	0	-	0.00	3	-	0.02	2	-	0.02	3	-	0.02	1	-	0.01	3		0.02			0.02
**Municipalities**																					
**Alvorada**	1	1	1.59	0	0	0.00	1	0	1.59	3	1	4.76	26	9	41.27	10	4	15.87	41	2	13.02
**Araguaçu**	13	6	21.89	3	1	5.05	9	3	15.15	5	2	8.42	9	3	15.15	5	2	8.42	44	3	12.35
**Araguaína**	159	70	12.25	134	69	10.32	214	63	16.48	155	60	11.94	203	69	15.64	201	70	15.48	1065	67	13.69
**Arapoema**	2	1	3.54	2	1	3.54	1	0	1.77	15	6	26.55	4	1	7.08	10	3	17.70	34	2	10.03
**Carmolândia**	3	1	14.22	1	0	4.74	1	0	4.74	6	2	28.44	1	0	4.74	2	1	9.48	14	1	11.06
**Colmeia**	1	0.5	1.50	19	10	28.57	26	8	39.10	10	4	15.04	7	3	10.53	1	0	1.50	64	4	16.04
Lagoa da Conf.**	5	2	4.50	9	5	8.10	38	11	34.20	33	13	29.70	25	9	22.50	42	15	37.80	152	9	22.80
**Lajeado**	1	0.5	3.79	3	1	11.36	3	1	11.36	6	2	22.73	6	2	22.73	6	2	22.73	25	2	15.78
**Nazaré**	31	14	80.94	21	11	54.83	46	13	120.10	27	10	70.50	12	4	31.33	10	3	26.11	147	9	63.97
**S^ta^ Terezinha** ***	9	4	43.90	3	2	14.63	2	1	9.76	0	0	0.00	0	0	0.00	0	0	0.00	14	1	22.76
**Other counties**	224	-	2.13	150	-	1.43	225	-	2.14	239	-	2.27	304	-	2.89	372	-	3.54	1415	-	2.40
**Microregions**																					
**Bico Papagaio**	67	19	3.45	45	13	2.32	67	12	3.45	45	9	2.32	35	6	1.80	30	5	1.55	289	10	2.48
**Araguaína**	196	56	8.03	183	53	7.50	274	48	11.23	182	36	7.46	251	42	10.29	287	43	11.76	1373	45	9.38
**Miracema**	6	2	0.51	25	7	2.14	29	5	2.49	19	4	1.63	30	5	2.57	18	3	1.54	127	4	1.81
**Rio Formoso**	21	6	2.18	13	4	1.35	68	12	7.06	49	10	5.09	50	8	5.19	65	10	6.75	266	9	4.60
**Gurupi**	6	2	0.57	4	1	0.38	9	2	0.85	16	3	1.51	46	8	4.34	38	6	3.59	119	4	1.87
**Porto Nacional**	21	6	0.72	59	17	2.03	81	14	2.79	138	28	4.76	151	25	5.20	181	27	6.24	631	21	3.62
**Jalapão**	29	8	3.90	13	4	1.75	23	4	3.09	35	7	4.71	25	4	3.36	23	3	3.09	148	5	3.32
**Dianópolis**	3	1	0.28	3	1	0.28	15	3	1.42	15	3	1.42	9	2	0.85	17	3	1.61	62	2	0.98

Rate: number of cases/population x 1000; AAR: average annual rate

*: ignored

**: Confusion

***: Santa Terezinha do Tocantins; %: horizontal = total and vertical between groups

**Fig 2:**
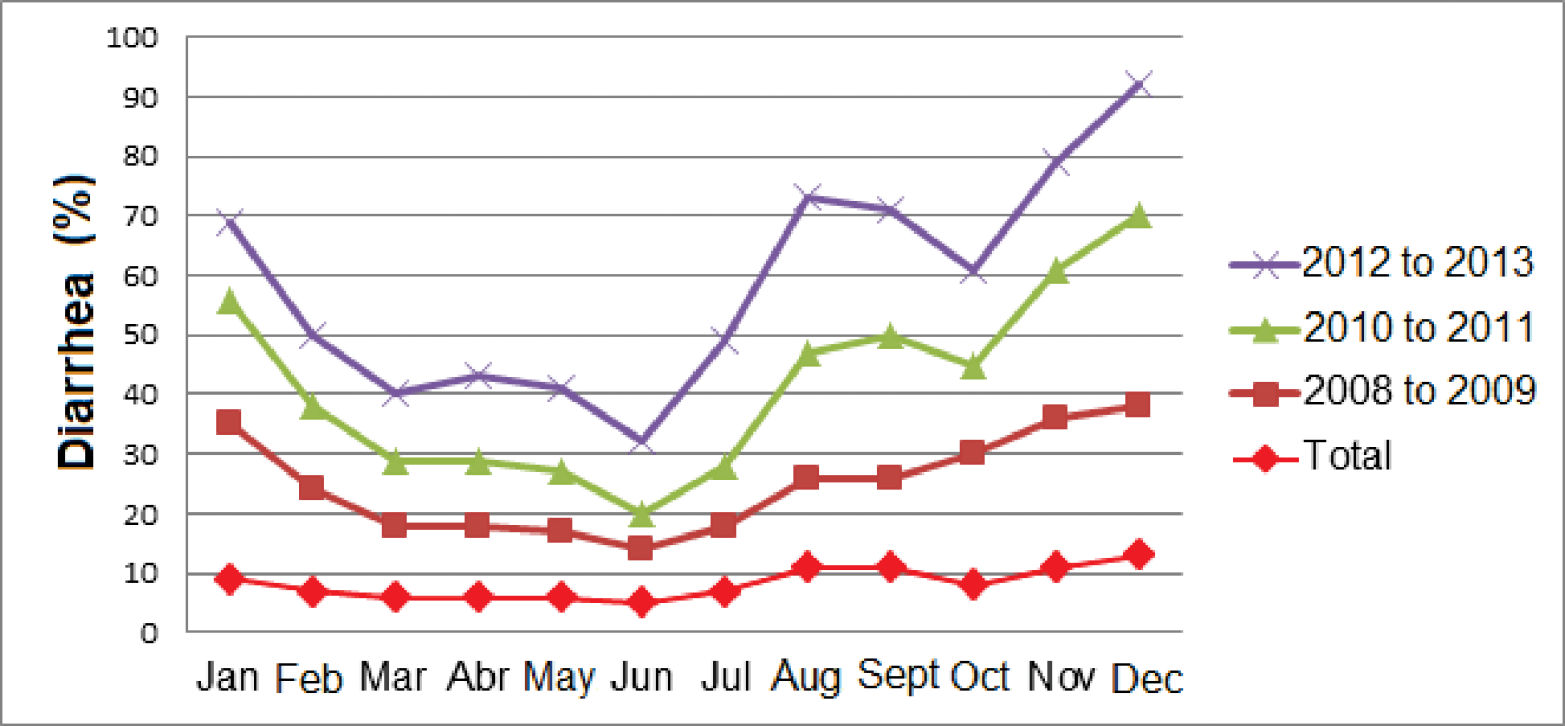
Percentage of hospitalizations for diarrhea in children aged zero to four full years, 2008 to 2013, according to the place of residence, Tocantins, Brazil.

Population characteristics were shown per year, between 2008 and 2013, according to the absolute and proportional number of cases (horizontal = total, vertical = within group) and rates (number of cases/population per group x 1,000 inhabitants), and by calculating the average annual rate (AAR) of each variable (Table 1). The percentage of children with diarrhea reported per month was shown in a line graph, according to the biannual distribution (2008 and 2009; 2010 and 2011; 2012 and 2013; total) (Fig 2). Data were calculated using Microsoft® Excel® 2016.

The TerraView *software* version 4.2.2 was used for the study of spatial statistics. This software was developed by the National Institute for Space Research (www.dpi.inpe.br/terraview/index.php). The Global Moran’s index and Local Moran’s index—LISA were used to evaluate spatial correlation and local autocorrelation, allowing the identification of subregions with spatial dependence. A first-order neighborhood criterion was used to make estimates, where neighbor counties were defined as those bordering each other [23,25]. The Global Moran’s index varies between -1 and 1; values nearing zero indicate no correlation, and values nearing 1 represent positive spatial dependence with greater similarity between neighboring counties (clustering) and negative spatial dependence is indicated as -1, indicating dissimilarity (dispersion). To evaluate the significance of the test, the criterion of 99 permutations [30] was used.

**Fig 3:**
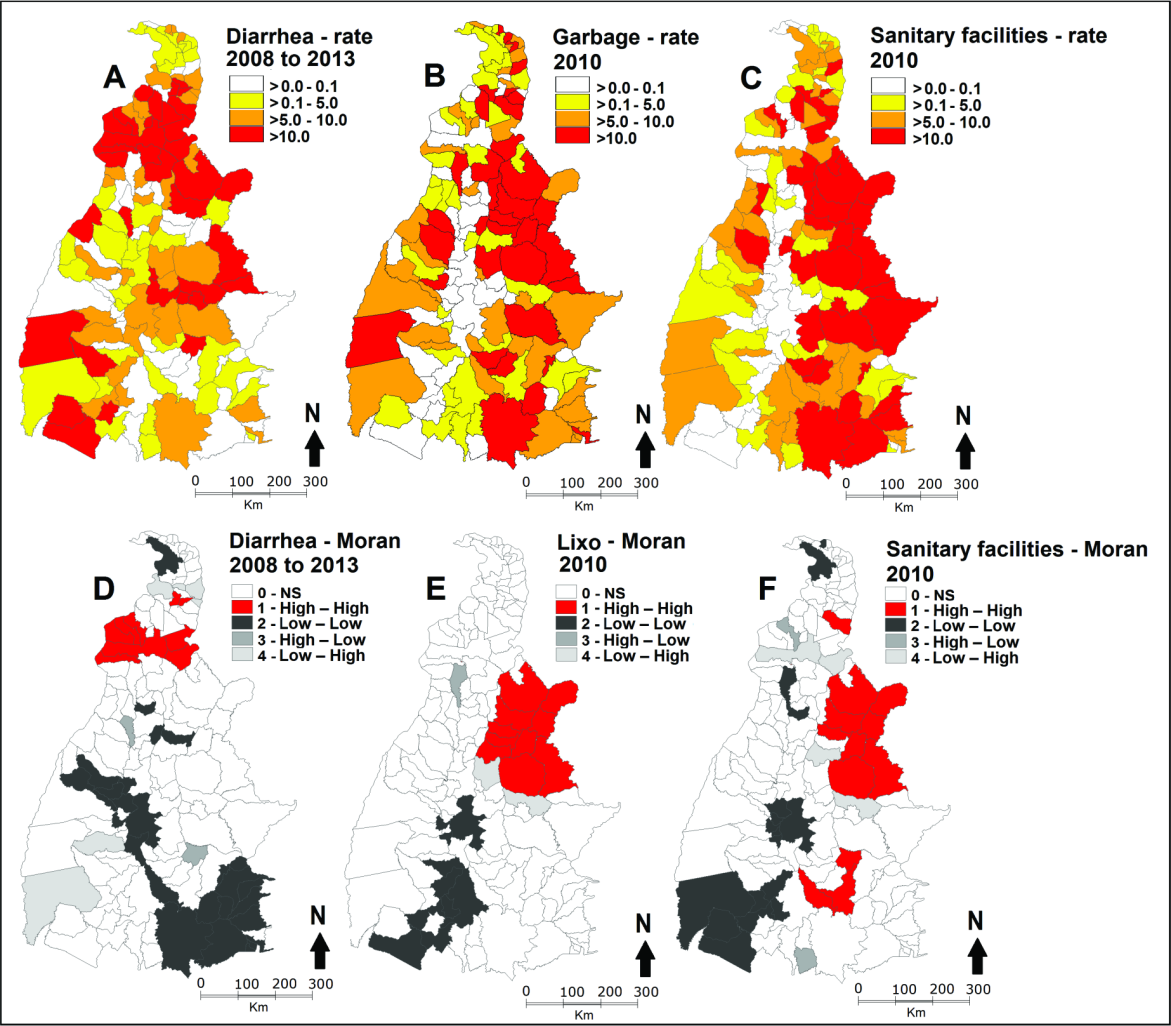
Maps for spatial analysis of the distribution of rates per 100,000 inhabitants, Tocantins, Brazil, and of the spatial distribution of the Global Moran’s index Map: A and D—hospitalization for diarrhea in under 5–year-old children by place of residence (2008 to 2013); B and E—counties with presence of garbage dumped on vacant areas and/or in the home environment (2010); C and F—counties without sanitary installations (2010).

Maps were constructed based on rates (number of cases/population x 100,000 inhabitants: data on diarrhea in under 5-year-old children, variables related to garbage "dumped on vacant areas and/or in the home environment" and counties "lacking sanitary installations" [25,31]. Results were defined in quantiles, which was the format that best represented the data, using the following intervals: > 0.0–0.1 (absence of cases or insignificant rate), > 0.1–5.0 (low rate), > 5.0–10.0 (intermediate rate), > 10.0 (high rate) (Fig 3). Thematic maps using different colors were prepared for better visualization of variation of rates [23].

Data for preparation of the Moran Map are only generated if any level of significance at the interface (> 95%) is detected, indicating priority locations for intervention [30]. The criterion was: "0"—white (not significant = NS); 1- red (Q1—high-high = high priority for intervention), positive values and negative means, with significance < 0.05; and 2—dark gray (Q2—low-low = low priority), negative rates and negative means—counties with positive spatial association or similar to neighbors; 3—medium gray (Q3—high-low = intermediate priority), positive values and negative means and 4—light gray (Q4—low-high = intermediate priority), negative values and negative means are considered counties with negative spatial association, that is, low and high rates of diarrhea, respectively [23,25]. It can be said that "1" and "2" represent areas of agreement and "3" and "4" areas of transition [23,25]. It is possible that random oscillations were minimized since 6 consecutive years were analyzed. All the figures in Results have granted permissions and were reprinted from INPE under a CC BY license, with permission from Dr. Lubia Vinhas, original copyright 2018.

### Ethics statement

Public domain secondary data found in databases and available on the *web* were used, and therefore there is no identification of individuals involved in the study. Thus, approval of the research by an Ethics Review Board was not necessary [32].

## Results

A total of 3,015 cases of hospitalizations for diarrhea in children aged zero to four full years, residents of the State of Tocantins, were reported in DATASUS in the studied period, 2008 to 2013. One hundred and seven out of 139 counties (77%) reported hospitalizations for diarrhea. The mean number of cases of hospitalizations for diarrhea was 502 per year, varying from 224 in 2008 to 672 in 2013, with an average annual rate (AAR) of 4.10 cases per 1,000 inhabitants (inhab.). Diarrhea was prevalent in males (55%); it had higher prevalence (75%) and incidence (128.02 per 1,000 inhab.) in brown-skinned people; it had a higher prevalence in the city of Araguaína (67%) but a higher incidence in the county of Nazaré (63.97/1,000 inhab.); it was prevalent and incident in the Araguaína microregion (45%, 9.38 per 1,000 inhab.) and had higher incidence in children under 1 year of age (7.75/1,000 inhab.). The AAR of deaths was 0.02 per 1,000 inhabitants. The Global Moran’s index was 0.11 (p < 0.03) (Table 1).

There was an increase in the number of hospitalizations among under 5-year-old children over the years, with lowest incidence in 2009 (2.81) and higher in 2013 (5.37), with a considerable increase among under 1-year-old children, with lower rate in 2008 (6.1/ 1,000 inhab.) and higher in 2012 (9.66/1,000 inhab.). The prevalence was higher among children aged 1 to 4 full years (varying from 58% to 70%).

The percentage of hospitalization for diarrhea in under 5-year-old children was higher between November and January and lower in June. However, the Fig 2 shows a high peak between the months of August and September, with a significant decrease between 2010 and 2013.

Hospitalization rates for diarrhea per 100,000 inhabitants were distributed on the thematic map (Fig 3) showing regions of clusters of counties in the Araguaína microregion, clearly indicating a spatial pattern and spatial correlation. The Moran index was 0.11 (p < 0.03).

The 10 counties with the highest rates (per 1,000 inhab.) of hospitalization for diarrhea in the state of Tocantins are shown in Fig 1 according to the following numbering, in decreasing order: Nazaré n° 19 (63.97/1,000 inhab.), Lagoa da Confusão N° 91 (22.80/1,000 inhab.), Santa Terezinha do Tocantins n^o^ 19 (22.76/1,000 inhab.), Colméia n° 59 (16.04/1,000 inhab.), Lajeado n^o^ 80 (15.78/1,000 inhab.), Araguaína n^o^ 35 (13.69/1,000 inhab.), Alvorada n^o^128 (13.02/1,000 inhab.), Araguaçu n^o^ 116 (12.35/1,000 inhab.), Carmolândia n^o^ 33 (11.06/1,000 inhab.), Arapoema n^o^ 40 (10.03/1,000 inhab.) (Table 1).

The Global Moran’s index presented significant positive values, giving evidence of spatial dependence among the rates of counties with similar patterns of diarrhea in under 5-year-old children, with index 0.11 (*p* < 0.03) (Fig 3D). The variables concerning the counties with garbage dumped on vacant areas and/or in the home environment had an index of 0.32 (*p < 0*.01) (Fig 3E); and the counties without sanitary installations had an index of 0.33 (*p < 0*.01) (Fig 3F).

In Fig 3 it is possible to observe clusters of counties based on the local Moran’s index for diarrhea rates between 2008 to 2013 (D); the variables of presence of garbage in 2010 (E), and counties without sanitary installations in 2010 (F). A large set of counties with high rates of diarrhea was identified in the Araguaína microregion (7 counties: Aragominas n^o^ 31, Mauricilândia n^o^ 32, Santa Fé do Araguaia n^o^34, Araguaína n^o^35, Pau D ' Arco n^o^37, Filadelfia n^o^ 36, Babaçulândia n^o^ 29) and a smaller cluster in the microregion of Bico do Papagaio (2 counties: Santa Terezinha do Tocantins n^o^22 and Angico n^o^20). Clusters with high rates (Q 1—high-high) of counties with no sanitary installations (12 counties) and with garbage dumped on vacant areas and/or in the home environment (10 counties) were similar, covering the same microregions, mostly of the Jalapão microregion, which borders the Araguaína microregion, and to the north of the Porto Nacional microregion.

Clusters of counties with low rates of diarrhea (Q 2—low-low) covered almost the entire Dianópolis microregion, north of the microregions of Gurupi and Rio Formoso, south of Miracema do Tocantins and Porto Nacional. Clusters of counties with low rates were mostly located in the southern mesoregion of the state. Absence of sanitary installations (17 counties) and garbage dumped on vacant areas and/or in the home environment (11 counties) were similar to the cluster of counties with low hospitalization rates for diarrhea of under 5-year-old children.

**Fig 4:**
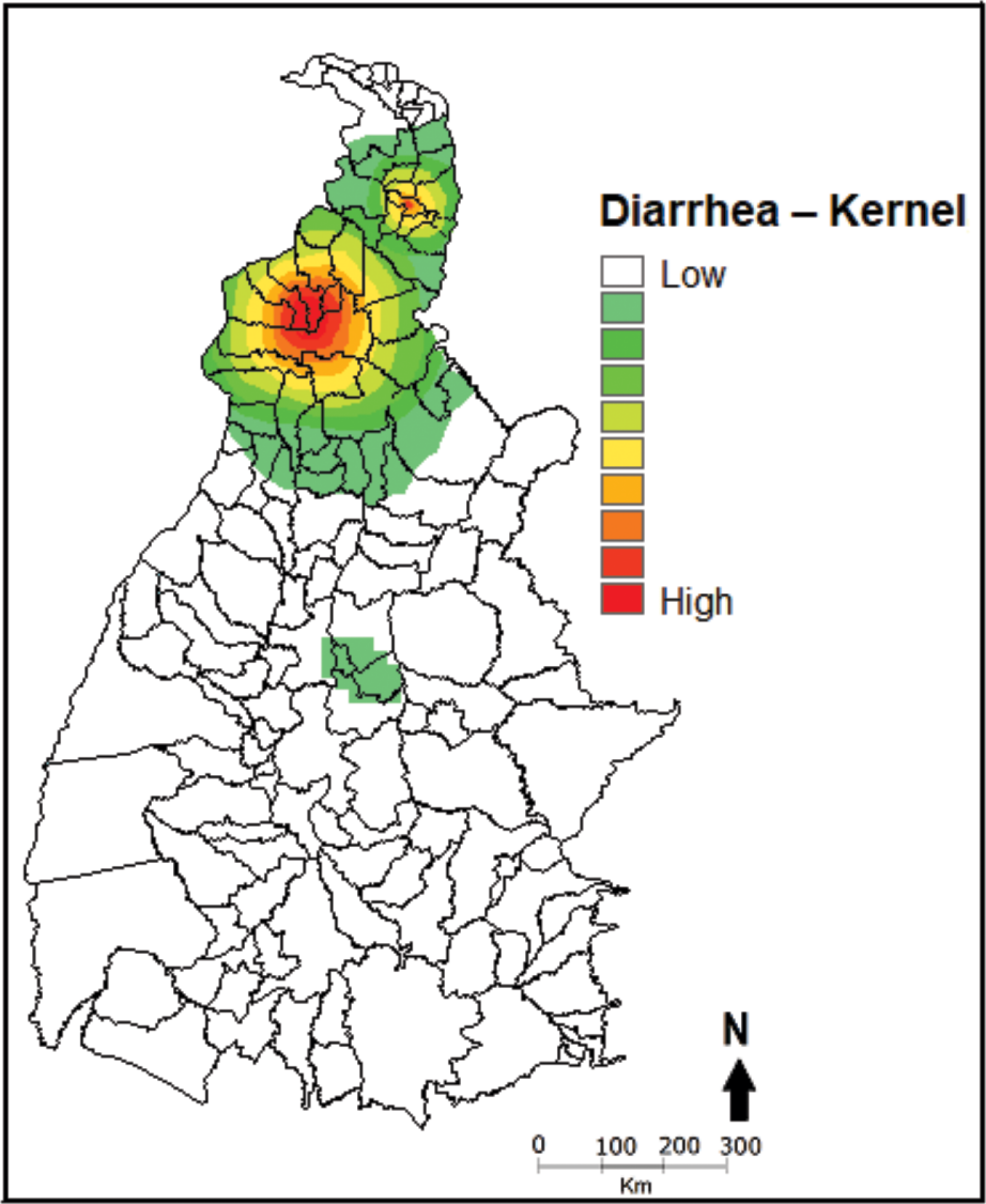
Kernel analysis of density of hospitalizations of children aged zero to four full years between 2008 and 2013, Tocantins, Brazil.

The Kernel density estimator (Fig 4) showed that the presence of red areas ("hotspots") in the northern region of the state, specifically in the Araguaína microregion, reveals the high density of counties that reported hospitalizations of younger children for diarrhea, according to the year of admission.

## Discussion

Populations in underdeveloped and in developing countries have faced problems related to environmental and climatic issues and poor quality of life, which are responsible for the increase of diarrheal diseases, besides other waterborne diseases [1–4,18]. Millions of people have suffered from diseases such as diarrhea, especially children, and this disease is one of the main causes of morbidity and mortality among children, especially in the group aged zero to four full years [9,11,14]. Most cases (50%) are caused by rotavirus in several countries [15,33], despite increased vaccination coverage, improved health conditions and knowledge about oral rehydration [5,9]. In Brazil, children aged up to 5 years are the most affected and 80% are younger than 1 year old [14,17], especially in the North region, where diarrheal diseases rank 8th in the causes of infant mortality [18]. During the first year of life, children are more vulnerable to environmental variations and conditions, especially when there is early weaning [34]. Immunity to rotavirus infections is usually developed after 1 or 2 infections, which usually occurs among children under 5 years of age. The rate of symptomatic infections drops considerably after this age [35].

This is the first study conducted in the state of Tocantins using spatial analysis tools with data on the incidence of hospitalizations for diarrhea in under 5-year-old children. Geoprocessing techniques made it possible to identify a spatial pattern of distribution of hospitalizations for diarrhea in under 5-year-old children, according to county, between 2008 and 2013. The data presented showed a cluster in the Araguaína microregion, with a positive autocorrelation between counties, with a Moran’s index of 0.11 and a statistical significance of *p* < 0.03. Fontoura; Fontoura and Nascimento (2016) carried out a spatial analysis in the Araguaína microregion and also detected a statistically significant cluster in the case of visceral leishmaniasis, a disease that also affects children in the first years of life [25]. From the 3,015 cases reported in 139 counties in the State of Tocantins, hospitalizations for diarrhea produced an average annual rate of 4.10 cases per 1,000 inhabitants. The Moran Map allowed identifying the higher and lower priority counties for intervention by competent authorities, in order to reduce cases of hospitalizations for diarrhea in the case of the counties belonging to "high-high" clusters [30,36].

Diarrhea was more frequent among male children (55%), similar to the findings of a survey conducted in Qatar, where 55.5% of diarrhea cases occurred in male children [12]. The hospitalization rate of children with diarrhea was higher in under 1-year-old children (7.75/1,000 inhabitants). Children younger than one year are more vulnerable to diarrheal diseases [14,17]. This fact may possibly be due to factors such as non-adherence to exclusive breastfeeding in the first six months of life [12,17]. This cause of hospitalization and death among children younger than one year is more frequent in the low-income and low HDI population [24]. Another factor is the lack of instruction of mothers, as well as the difficulty of accessing health services [2,9–12]. The percentage of women with positive rotavirus infection was high in a study conducted in India, suggesting that this can be due to their role as caregivers [35], likely representing a source of infection for children.

Diarrhea has multiple pathways of transmission [5], but the most common is untreated contaminated water coupled to the low availability of this resource [9,18] and poorly built houses with a lack of basic infrastructure [10,20]. The main causes of infection are social inequality, poor hygiene, low socioeconomic status, overpopulation, low birth weight, low level of knowledge of mothers, lack of adherence to breastfeeding, poor conditions and difficult access to public services [2,3,9–12], with increased rates after hydrometeorological catastrophes and events such as floods [18], which may lead to malnutrition [17]. The probability of infections among adults and adolescents is high, and may be symptomatic or asymptomatic depending on the host's immunity, with a possible close association with transmitters of germs causing diarrhea [35].

The increased number of hospitalizations reported in the period of 6 years can be attributed to underreporting in previous years. One of the problems detected when using geoprocessing tools allied to official data is underreporting or lack of complete information [10]. Other probable factors that increase the number of notifications may be the better access to curative health measures in detriment to preventive measures, what leads populations to seek hospital services more often [2,3,9–12], or when the disease is already severe, requiring hospitalization for its treatment. A review showed that the introduction of a highly effective program to combat infectious diseases considerably reduced infant mortality, but had no effect on growth [19].

It was possible to observe the presence of clusters with high rates in the North of the state of Tocantins with respect to cases of diarrhea in under 5–year-old children and also to the variables related to counties with garbage dumped on vacant areas and/or in the home environment and without sanitary installations. There were also clusters with low rates in the south of the state. Poorly built households with lack of adequate infrastructure, without sanitary facilities, garbage collection, and with contaminated water have led to transmission of diarrheal diseases [2,3,9–12]. Good basic sanitation and adequate garbage collection helps to maintain health and, consequently, to improve the living conditions of inhabitants. The quantity and quality of the available water are important factors for maintenance of health and are related to the reduction of the incidence and prevalence of various diseases such as diarrhea diseases [37]. The exposure of children to poor sanitation and hygiene are the main causes of the so-called environmental enteric dysfunction [19].

Many difficulties permeate spatial studies because official data such those available in the SINAN are not always complete due to underreporting [31,36]; little information or incomplete data may leave researchers without denominators to compute the rates and the independent variables to explain the spatial causes possibly associated with morbidity and mortality [10,38]. The techniques used in spatial analysis tools still need to be explored in order to improve their potential to benefit health services [26].

This study may have limitations including diagnostic errors, lack of diagnosis of the pathogen (non-available on DATASUS) of cases of diarrhea since medical records were not examined; lack of information on housing conditions (non-available data), water supply and garbage collection. Another possible limitation may be associated with a lack of information on the frequency of each hospitalization, that is, whether it was the case of a first hospitalization for diarrhea or recurrent hospitalization.

However, it was possible to identify clusters of cities with high rates of hospitalization for diarrhea, providing subsidies for municipal managers to search out the possible causes.

With this study, it was possible to identify spatial patterns in the distribution of diarrhea in the state of Tocantins, Brazil, through thematic maps where hotspots for intervention of public authorities and health agents were identified. The density of rates was also identified for counties of the state. These findings have implications in terms of public health and call for planning of intervention actions aimed at the prevention and control of diseases such as infant diarrhea. The results also endorsed that good sanitary installations, adequate garbage collection, access to health services and health education actions can promote better living conditions and health for the population, as well as prevent diseases such as diarrhea.
